# Effect of an Atmospheric Pressure Plasma Jet on the Structure and Physicochemical Properties of Waxy and Normal Maize Starch

**DOI:** 10.3390/polym11010008

**Published:** 2018-12-21

**Authors:** Yaping Zhou, Yizhe Yan, Miaomiao Shi, Yanqi Liu

**Affiliations:** 1 School of Food and Biological Engineering, Zhengzhou University of Light Industry, Zhengzhou 450002, China; ZYP4265@163.com (Y.Z.); chengzi3090@126.com (M.S.); 2 Henan Collaborative Innovation Center of Food Production and Safety, Zhengzhou 450002, China; 3 Henan Key Laboratory of Cold Chain Food Quality and Safety Control, Zhengzhou 450002, China

**Keywords:** atmospheric pressure plasma jet, maize starch, modification, structure, property

## Abstract

In present study, a novel physical modification of waxy maize starch (WMS) and normal maize starch (NMS) was investigated by using an atmospheric pressure plasma jet (APPJ) treatment. The effect on the structure and physicochemical properties of both starches was demonstrated by treatment with a 5% starch suspension (*w*/*w*) with APPJ for short periods of time (1, 3, 5, or 7 min). The pH of WMS and NMS was decreased after APPJ treatment from 5.42 to 4.94, and 5.09 to 4.75, respectively. The water-binding capacity (WBC) (WMS: 105.19%–131.27%, NMS: 83.56%–95.61%) and swelling volume (SV) (WMS: 2.96 g/mL–3.33 g/mL, NMS: 2.75 g/mL–3.05 g/mL) of the starches were obviously increased by APPJ treatment. The surfaces of starch granules were wrecked, due to plasma etching. No changes in the crystalline types of both starches were observed. However, the relative crystallinities (RCs) of WMS and NMS were reduced from 46.7% to 42.0%, and 40.1% to 35.7%, respectively. Moreover, the short-range molecular orders of both starches were slightly reduced. In addition, APPJ treatment resulted in lower gelatinization temperature and enthalpies. Therefore, APPJ provides a mild and green approach to starch modification, showing great potential for applications in the food and non-food industry.

## 1. Introduction

Starch is a natural carbohydrate, and a primary source of both stored energy and reduced carbon in most plant species. Starch is not only the main component of food, providing a vital energy for humans, but is also widely used in the non-food industries, such as paper, textiles, bioethanol, cosmetics, etc. [1,2,3,4]. Starch is a carbohydrate that is formed by glycosidic linkages between glucose units, which are basically comprised of two fractions: amylose and amylopectin. Amylose is mostly a linear polymer with α–1,4–D glucopyranose units, and amylopectin is a highly branched molecule with α–1,4–D glucopyranose units in chains by α–1,6 glucopyranose linkages [5]. Despite the starch being widely used as mentioned above, the inherent characteristics of natural starch, such as high viscosity, turbidity, and instability, limit the applications for use [6]. Thus, the modification of starch becomes particularly important. At present, there are a few modifying methods, including physical, chemical, and enzymatic modifications [7,8]. In general, the chemical modification involves acid hydrolysis, oxidation, etherification, esterification, and cationization, but these methods result in large chemical pollution [9]. The enzymatic modification refers to the treatment of starch with various enzymes, such as glucoamylase, and debranching enzyme. In spite of the good specificity, this method requires expensive enzymes and strict enzymolysis conditions [10]. Furthermore, the physical modification of starch mainly includes hydrothermal, ultrasonic, ultra-high pressure, and plasma treatment, in which only water and energy are involved without extra reagents. Therefore, it has emerged as an important method for clean production and green food processing [11,12,13,14]. Although great progress on starch modification have been made in recent years, a safe and efficient modification is highly desirable.

Plasma is considered to be the fourth state of matter in addition to gases, liquids, and solids [15], and contains many active species, such as electrons, ions, free radicals, excited atoms, and generous neutral molecules [16]. Plasma processing as an emerging novel non-thermal physical technology [17], is mainly used for microbial and enzymatic inactivation [18,19], enhancement of the rate of germination of seeds [15], starch modification [20], reduction in the cooking time of rice [21], and improvement of plastics properties [22]. According to the plasma generation apparatus, it can be divided into the dielectric barrier discharges, corona glow discharges, radio frequency, and gliding arc discharge, etc. [16]. According to the difference of plasma feed gas, it can be divided into air, oxygen, ammonia, and so on [23]. Plasma, as a green technology to change material characteristics, has attracted much attention in starch modification in recent years. The influence of plasma on starch depends on the plasma generation apparatus, and the type of feed gas, as well as the treatment time and the voltage [19]. Although the method of plasma modification is different, the plasma modification of starch is mainly involved in three mechanisms, including cross−linking, depolymerization, and etching [16].

Recently, many scientists have employed plasma in various methods of modifying starches with changes in structure and properties [14,20,24,25]. Nevertheless, in most studies, dry starch powder was directly treated with plasma with unsatisfactory uniformity and efficiency. Therefore, a new treatment method by plasma is highly required. In this study, a novel physical modification of waxy maize starch (WMS) and normal maize starch (NMS) was developed, by treating the starch suspension with APPJ. The changes of the structure and the physicochemical properties of WMS and NMS were investigated after APPJ treatment.

## 2. Materials and Methods 

### 2.1. Materials

Waxy maize starch (WMS) was purchased from Hengrui starch Import & Export Co. Ltd. (Luohe, China), with 12.1% moisture and 4.5% amylose. Normal maize starch (NMS) was purchased from Lihua starch Import & Export Co., Ltd. (Qinhuangdao, China) with 10.9% moisture and 23.2% amylose. The reagents used here were of analytical grade (ethanol, etc.).

### 2.2. Exposure of Starch Suspension to APPJ

The WMS and NMS (5.0 g) were added to deionized water (100 mL) to prepare a slurry (5% *w*/*v*). The slurry was thoroughly mixed by using a vortex mixer (2000 rpm, 10 min) before treatment.

**Figure 1:**
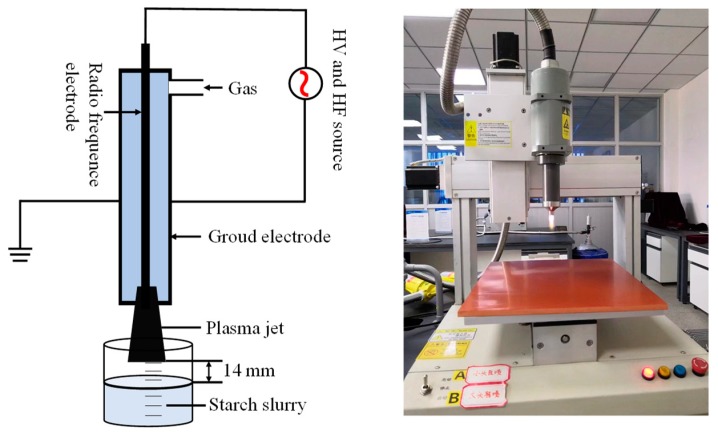
The schematic and device diagram of the atmospheric pressure plasma jet (APPJ) apparatus.

The starch slurry was placed in test tube (height: 12 cm, diameter: 2.7 cm) with an APPJ treatment at 750 W of the input power supplied; the HF of power supply was 25 kHz, and H = 14 mm (distance from plasma jet probe to sample). The schematic diagram of APPJ (Eastcom Gaoke Automation Equipment Co., Ltd, Shenzhen, China) setup was shown in Figure 1. APPJ was induced by supplying sufficient input power and high frequency under atmospheric pressure. Under the action of high voltage, the gas was ionized to generate plasma when passing through the gap between the two coaxial electrodes. During the action of the gas pressure, the plasma was ejected from the spout, and contacted with the starch suspension. The starch slurry was treated by APPJ for 1, 3, 5, or 7 min, respectively. The resulting solution was centrifuged (3000 rpm, 6 min) to obtain a precipitate, and washed with absolute ethanol three times. Finally, the precipitate was lyophilized in a vacuum freeze dryer and collected to obtain the starch samples.

### 2.3. pH of Starch Suspension

The pH of untreated or treated WMS and NMS was estimated for 0.5% (*w*/*v*) aqueous solutions according to Annapure’s method [25].

### 2.4. Water Binding Capacity (WBC) and Swelling Volume (SV)

The WBC of the starch samples was determined by using Cristina’s method with minor modifications [26]. WBC was defined as the water retention of a sample at low speed centrifugation. The samples (1.000 ± 0.005 g) were mixed with distilled water (10 mL), and centrifuged (2000 rpm, 20 min). The WBC was calculated by dividing the water-holding weight of the starch by the initial dry weight of the sample. The WBC of starch was analyzed in triplicate.

The SV of the starch samples was determined, following the method reported by Gularte’s and Rosell’s, with slight modification [27]. The samples (1.000 ± 0.005 g) were placed in a graduated centrifuge tube with added deionized water (10 mL). The mixture was thoroughly mixed using a vortex mixer (2000 rpm, 5 min) and equilibrated for 24 h at room temperature. The volume of water retained per gram of solid was calculated. The SV of starch was analyzed in triplicate.

### 2.5. Scanning Electron Microscopy (SEM)

The morphology of starch samples was imaged using a JSM-6490LV scanning electron microscope (PhilipsXL−3, Rili Co. Ltd., Tokyo, Japan). Double-sided carbon tape was used to fix the starch samples onto the test metallic platform, and gold was sprayed for coating (120 s) using a sputter coater (Polaron Sputter Coat System, Model 5001, Quorum Co. Ltd., East Sussex, UK). An accelerating voltage of 25 kV was used for imaging [13]. Micrographs of representative granules were taken, and the selected image was used at magnifications of 1000× or 5000×, respectively.

### 2.6. X-Ray Diffraction (XRD)

XRD analysis was performed using a Bruker D8 ADVANCE X-ray diffractometer (Bruker Co. Ltd., Karlsruhecity, Germany) operating at 40 kV and 30 mA. The starch samples (about 0.5 g) were placed in a circular test plate (diameter 22 mm, thickness 1.5 mm) and pressed. According to Wang’s method [28], the starch samples were equilibrated over a saturated sodium chloride (NaCl) solution at room temperature for one week before analysis. Other test conditions included a scan area of 5°–35° (2θ), a scanning speed of 2°/min, a step size of 0.02°, and one repetition [13]. The RC was quantitatively estimated as a ratio of the crystalline area to the total area between 5°–35° (2θ) using Origin software (Version 7.0, Microcal Inc., Northampton, MA, USA).

### 2.7. Fourier-Transform Infrared Spectroscopy (FT−IR)

FTIR spectra of the starch samples was measured using a Vertex 70 FTIR spectrometer (Bruker Co. Ltd., Karlsruhe, Germany) equipped with a Deuterated triglycine sulfate (DTGS) detector. The dried KBr was fully ground and mixed with the dried samples at a percent of 1% (*w*/*w*), and tablets approximately 1 mm thick were prepared. The absorbance spectra was computed between 4000 and 400 cm^−1^ with a resolution of 4 cm^−1^. The air was used as a blank, with a total of 64 scans for each sample at room temperature [13]. All FTIR-measured data were analyzed using OMNIC 8.2 software. The spectrum data were deconvoluted in the region of 800–1200 cm^−1^ and normalized. The ratios of absorbance at 1047/1022 cm^−1^ were used to estimate the short-range ordered structure of starch [11]. All measurements were performed in triplicate.

### 2.8. Raman Spectroscopy

Raman spectra of starch samples was obtained by using a BWS465-785S manual portable Raman spectrometer (B&W TEK Co. Ltd., Newark, DE, USA). A minor amount of starch was placed in a test groove and pressed. Spectra was taken from in the range of 3200–100 cm^−1^, the resolution was 4.5 cm^−1^, the integral time was 10,000 ms and the laser power was 100 mW. The dark currents were subtracted before testing [11]. The full width at half height (FWHH) of the band at 480 cm^−1^ was calculated by using the software of BWIQ. All measurements were performed for over three times to obtain the stable spectra.

### 2.9. Differential Scanning Calorimetry (DSC)

The gelatinization characteristics of the starch samples were characterized using a Q20 DSC (TA Co. Ltd., Newcastle, DE, USA). The baseline and furnace temperature were calibrated using indium, prior to testing. Nitrogen was used as purge gas at the rate of 15 mL/min. Starch samples (3.0 mg) were weighed directly in an aluminum pan, and distilled water was added to approximately 10.0 mg. The pan was hermetically sealed and allowed to equilibrate for 12 h at room temperature. The sample pans were heated from 30 °C up to 120 °C at the rate of 10 °C/min [29]. A sealed empty pan was used as a reference. The gelatinization temperatures of starches were determined by TA 2000 analysis software.

### 2.10. Statistical Analysis

Results were reported as the mean values and standard deviations of at least duplicate measurements three times. An analysis of variance (ANOVA) by Duncan’s test (*P* < 0.05) were conducted using the IBM SPSS Statistics 22.0 Software Program (SPSS Inc. Chicago, IL, USA).

## 3. Results and Discussion

### 3.1. pH, Water Binding Capacity, and Swelling Volume

There was a significant decrease in the pH of starches after APPJ treatment (Table 1). As the treatment time of APPJ increased, the pH of the treated starch gradually decreased. The pH of WMS and NMS was decreased from 5.42 to 4.94, 5.09 to 4.75, respectively. Similar results were obtained by Banura et al. [25] and Thirumdas et al. [29]. Plasma treatment was generally caused by the acidification of the starch, which was mainly related to some of the nitrogen-containing active substances produce by APPJ treatment, such as NO_2_^−^, NO_3_^−^, etc. The changes in pH might be due to oxidation of the starch granular structure, caused by the reactive species of plasma [16,30].

WBC of the starch was obviously increased by APPJ treatment (Table 1). As the treating time of APPJ increased, the WBC of WMS was continuously increased from 105.19% to 131.27%. Similarly, the WBC of NMS was increased from 83.56% to 95.61%. Banura et al. [25] also found the WBC of corn and tapioca starches was increased by plasma treatment. This increase might be caused by two reasons, one is the formation of simple sugars, such as glucose and maltose, via the depolymerization of plasma, and the other one is granular corrosion by plasma etching [17,25]. SV of WMS was found to be improved after the APPJ treatment. The SV of untreated WMS was 2.96 g/mL, and improved to 3.33 g/mL after plasma treatment for 7 min. A similar change in SV was also observed for APPJ-treated NMS, of which the SV increased from 2.75 g/mL to 3.05 g/mL. In previous studies, some analogous increases in SV were found by Pal et al. [31] and Sarangapani et al. [32]. The increased in SV might be due to the increase of hydrophilic groups. It is noteworthy that APPJ treatment resulted in a larger effect on pH, WBC, and SV of WMS than NMS.

### 3.2. Granular Morphology

**Figure 2:**
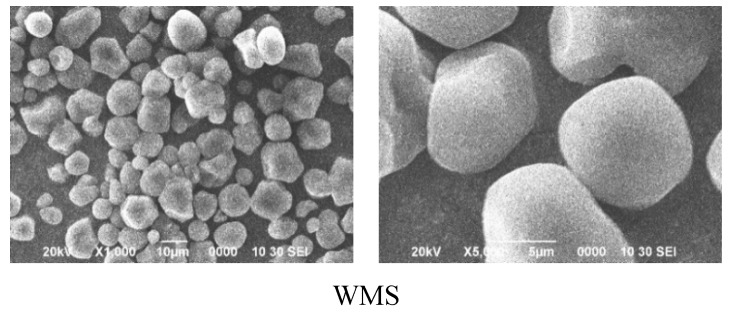

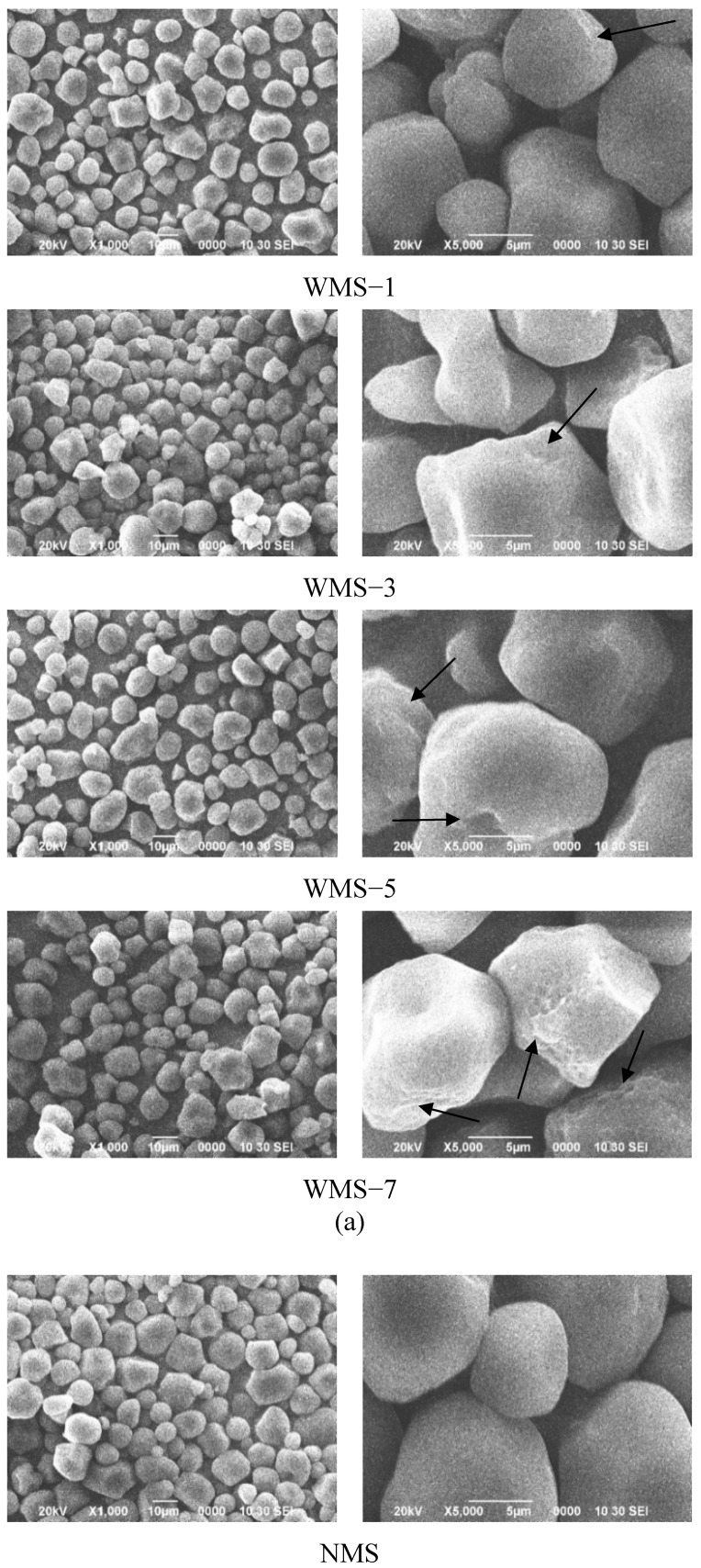

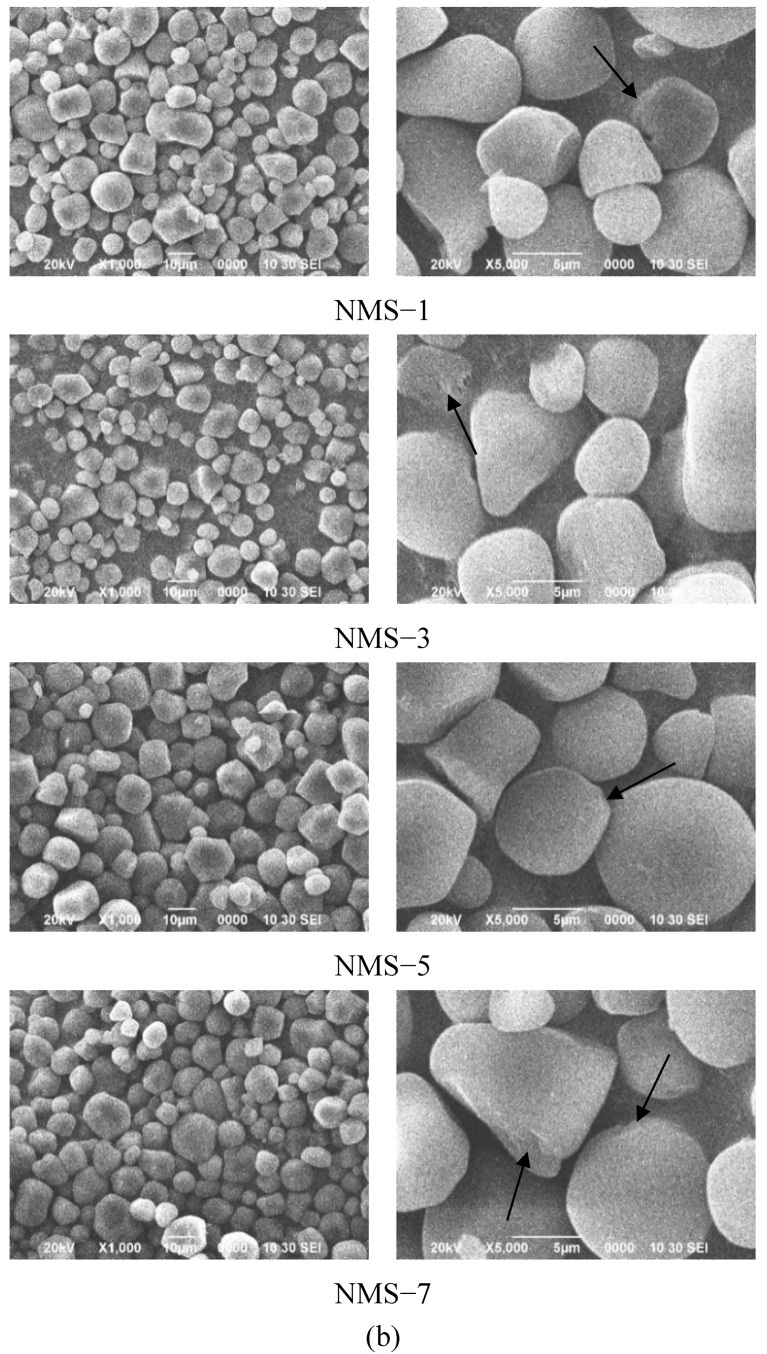
SEM images of maize starch granules treated by APPJ. (**a**) Waxy maize starch (**b**), normal maize starch.

The granular morphology of WMS and NMS granules was presented in Figure 2. All of the starch granules showed a mixture of the angular granule with a distinct non-uniformity in size [33]. There was obvious etching appearance on the surface of starch granules after APPJ treatment. This appearance was more distinct with an increase of the treating time of APPJ, as labeled by the arrows. Similar effects of plasma etching on starch granule morphology were also reported by other researchers [16,21,24,30]. The plasma etching might increase the surface energy of the granules, which is one of the important reasons for the increasing in hydrophilic nature [25]. The etched surface made water molecules easily convert into starch granules, which resulted in changes of the hydrophilic and pasting properties of starch [16]. In this study, the etching of APPJ might result in the increases of WBC and SV for all treated starches.

### 3.3. Crystalline Structure

**Figure 3:**
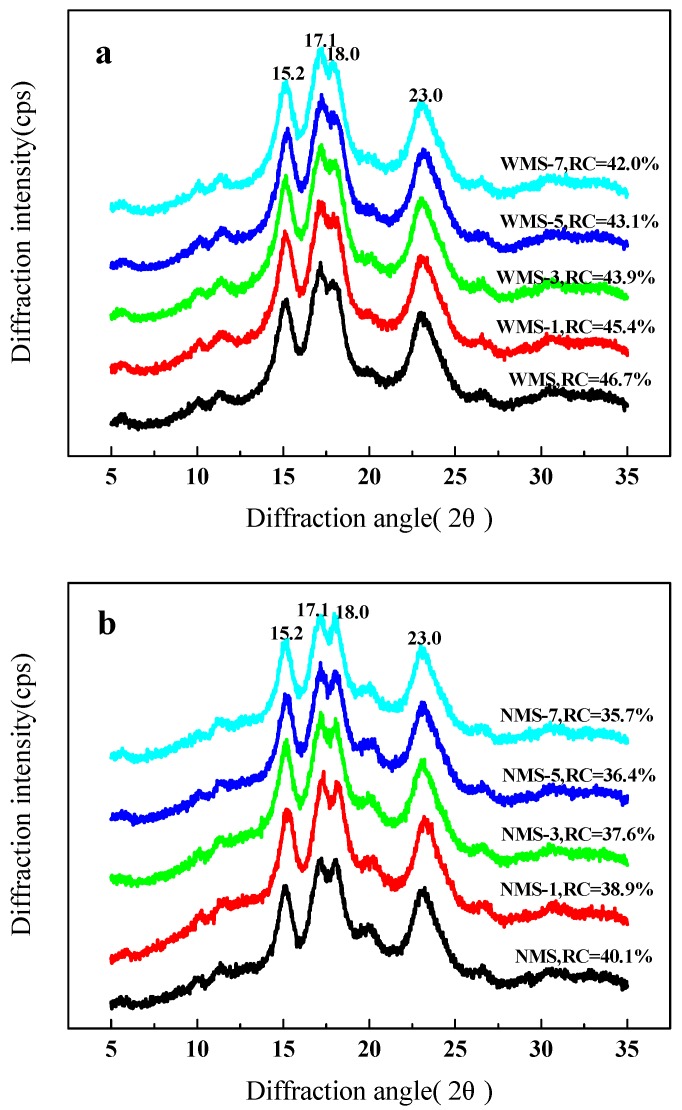
XRD patterns of maize starch samples treated by APPJ. (**a**) Waxy maize starch (**b**), normal maize starch.

The type of crystallinity pattern can be distinctly revealed by the XRD analysis. The diffraction characteristic peaks of the starch were displayed at 15.2°, 17.1°, 18.0°, and 23.0° (Figure 3), which was a typical diffraction pattern of the A–type crystallization [34]. There was no effect on the change of the crystallization type of WMS and NMS by APPJ treatment.

The same results have been reported in previous studies [23,29]. However, the peak intensity and the RC were slightly decreased, with treatment time increasing. The increase of APPJ treatment time decreased the RC of WMS from 46.7% to 42.0% (Figure 3a). Similarly, the RC of NMS was also decreased from 40.1% to 35.7% (Figure 3b). Thirumdas et al. [29] observed a similar decrease in the RC of rice starch after cold plasma treatment. Bie et al. [20] also found that the crystalline structure of corn starch granules using X-ray photoelectron spectroscopy was destroyed by dielectric barrier discharge plasma treatment. The decrease of crystallinity could be attributed to the interactions of the reactive species of plasma with the starch molecules, such as depolymerization [23,24,26,35].

### 3.4. Molecular Structure as Determined by FTIR

**Figure 4:**
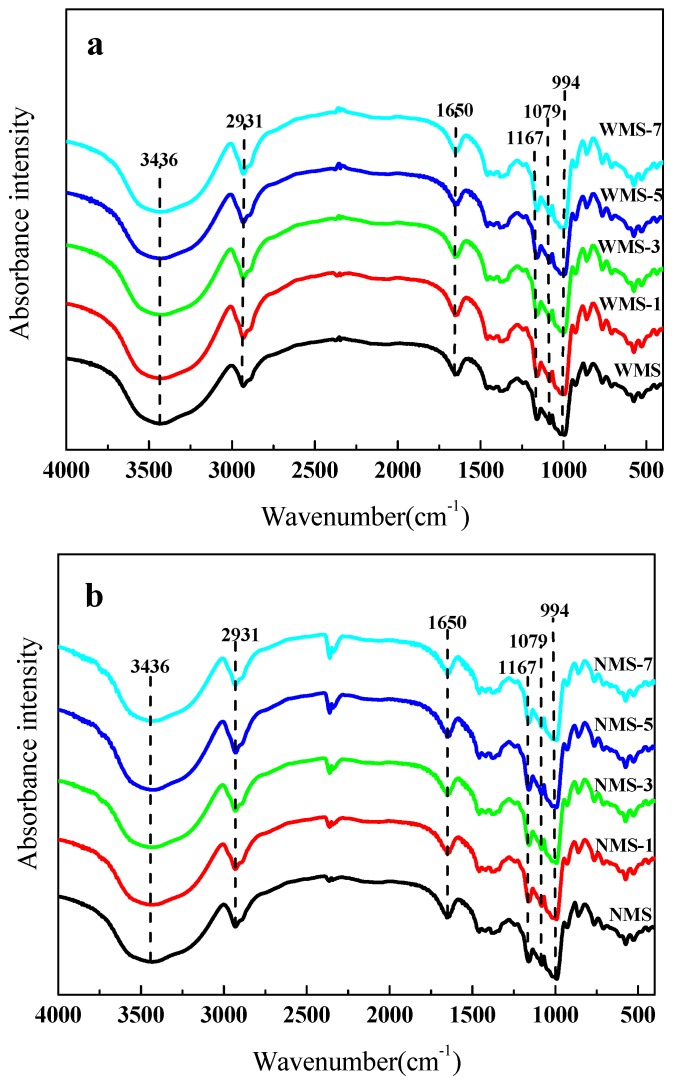
Fourier-transform infrared spectroscopy (FTIR) spectra of maize starch samples treated by APPJ. (**a**) Waxy maize starch (**b**), normal maize starch.

The molecular structure characterization of WMS and NMS by APPJ treatment was studied by FTIR, as shown in Figure 4. The width peak at 3436 cm^−1^ was attributed to the –OH group telescopic vibrational mode of starch [36]. A slight improvement of the O–H groups was found at the absorption peak at 3436 cm^−1^ (Figure 4). Thirumdas et al. [29] and Deeyai et al. [36] also demonstrated an increase of the absorption peaks of O–H groups during the plasma treatment of starch. This might be due to the depolymerization of starch glycosidic bonds caused by plasma species [19]. The infrared absorption at 2931 cm^−1^ and 1650 cm^−1^ was associated with the C–H deformation of the glucose unit, and the bending vibration of the –OH of water absorbed in the amorphous regions of starch, respectively [37]. The absorption peaks at 1167 cm^−1^, 1079 cm^−1^, and 994 cm^−1^ corresponded to the asymmetric C–O–C, C–O, and C–O–H skeleton stretching vibrations, respectively [38].

**Figure 5:**
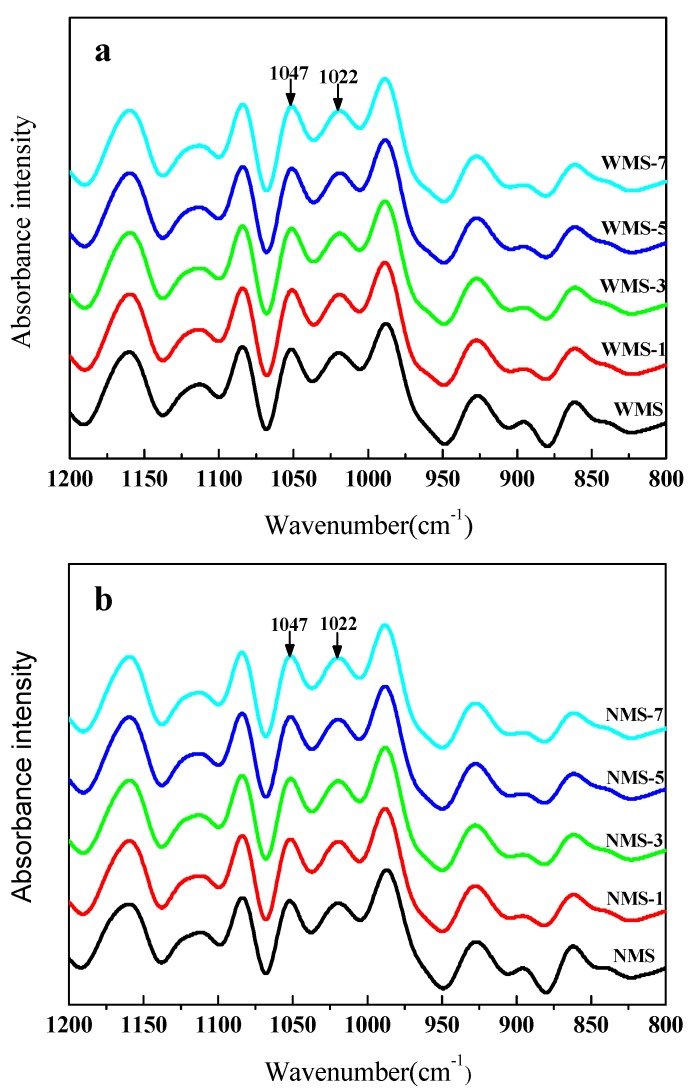
Deconvoluted FTIR spectra of maize starches from 1200–800 cm^−1^. (**a**) Waxy maize starch (**b**), normal maize starch.

To investigate the changes in the short-range molecular order of two maize starches before and after treatment by APPJ, the FTIR spectra of starch in the range of 1200–800 cm^−1^ were deconvoluted (Figure 5). The FTIR bands at 1047 cm^−1^ and 1022 cm^−1^ have been shown to be associated with crystalline and amorphous structures of starch, respectively [39]. The ratio of absorbances at 1047/1022 cm^−1^ could be used as the indexes of the short-range order of starch molecule double helices [39,40,41]. After the plasma treatment, a slight decrease in the value of 1047/1022 cm^−1^ was observed for both maize starches (Table 1), from 1.0549 to 1.0472, and 1.0311 to 1.0197, respectively. This suggested that the short-range order of starch was slightly reduced by the APPJ treatment. Bie et al. [20] also observed a similar decrease of the ratio of absorbances at 1047/1022 cm^−1^ for corn starch after dielectric barrier discharge plasma treatment. The changes might be due to the depolymerization of plasma. The depolymerization might dominantly occur in cleaving the glycosidic bonds of the starch molecule, which further changed the short-range order of the starch molecule. The ratio at 1047/1022 cm^−1^ of the APPJ-treated samples was slightly different from that of the untreated starch samples, indicating the mild modifications in the short-range order of starch molecule double helices.

### 3.5. Molecular Structure Determined by Raman

**Figure 6:**
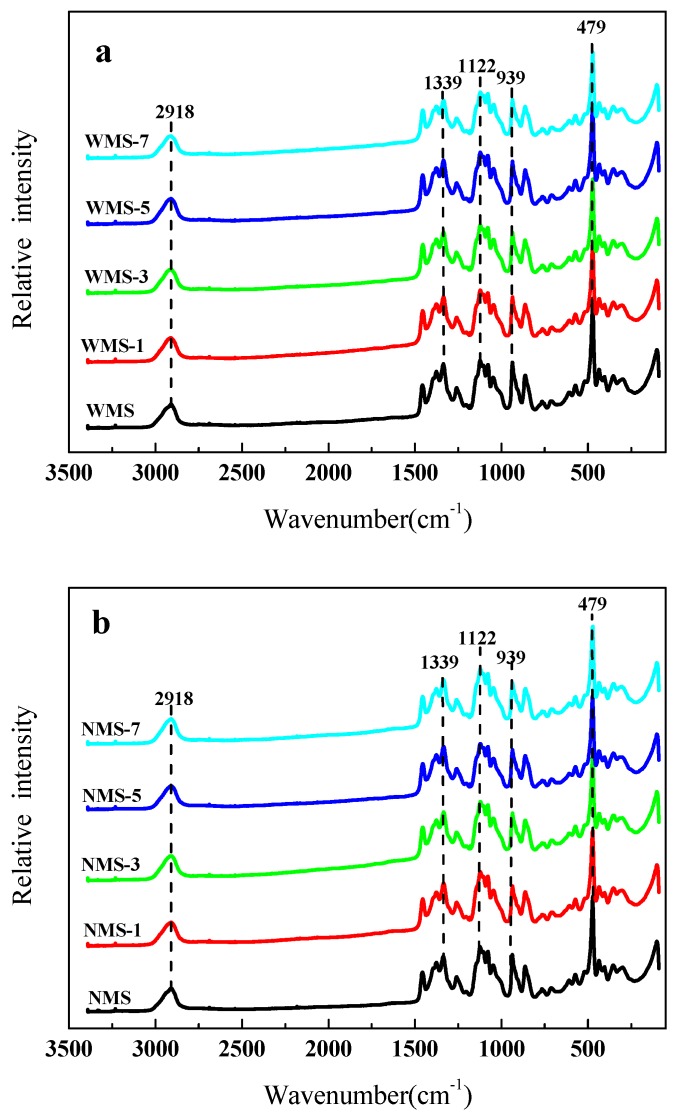
Raman spectra of maize starch samples treated by APPJ. (**a**) Waxy maize starch (**b**), normal maize starch.

Raman could be used to obtain information about the molecular structure of starch. The Raman spectra of both maize starches for treated and untreated by APPJ was presented in Figure 6, respectively. Several clear spectra bands could be seen at 2918 cm^−1^, 1339 cm^−1^, 1122 cm^−1^, 939 cm^−1^, and 479 cm^−1^. The bands at 2918 cm^−1^ and 1339 cm^−1^ were related to C–H stretching and C–O–H bending. The band at 1122 cm^−1^ was attributed to C–O stretching and C–O–H deformation, and the band at 939 cm^−1^ could be attributed to the C–O–C skeletal mode vibrations of the α–1,4 glycosidic linkages. Moreover, the 479 cm^−1^ was mostly caused by vibration of the skeleton of the glucose pyran ring [38,42,43,44]. A slight decrease of peak intensity was found in the Raman spectra after APPJ treatment.

The FWHH of 479 cm^−1^ could be also widely used to characterize the short-range order of starch samples [37,42,45]. In general, the FWHH of starch samples was inversely proportional to the ordered structure [46]. The FWHH of WMS by APPJ treatment was slightly changed (Table 1). The FWHH was slightly increased from 16.96 to 17.60 cm^−1^ with a prolonged plasma treatment. Similarly, the FWHH of NMS was slightly improved after treatment by APPJ. However, no significant effect was observed with the increase of plasma treatment time. Raman results suggested that the short-range order of starch was slightly reduced by the APPJ treatment [11,37,43], which was consistent with the FTIR results.

### 3.6. Gelatinization Properties

**Figure 7:**
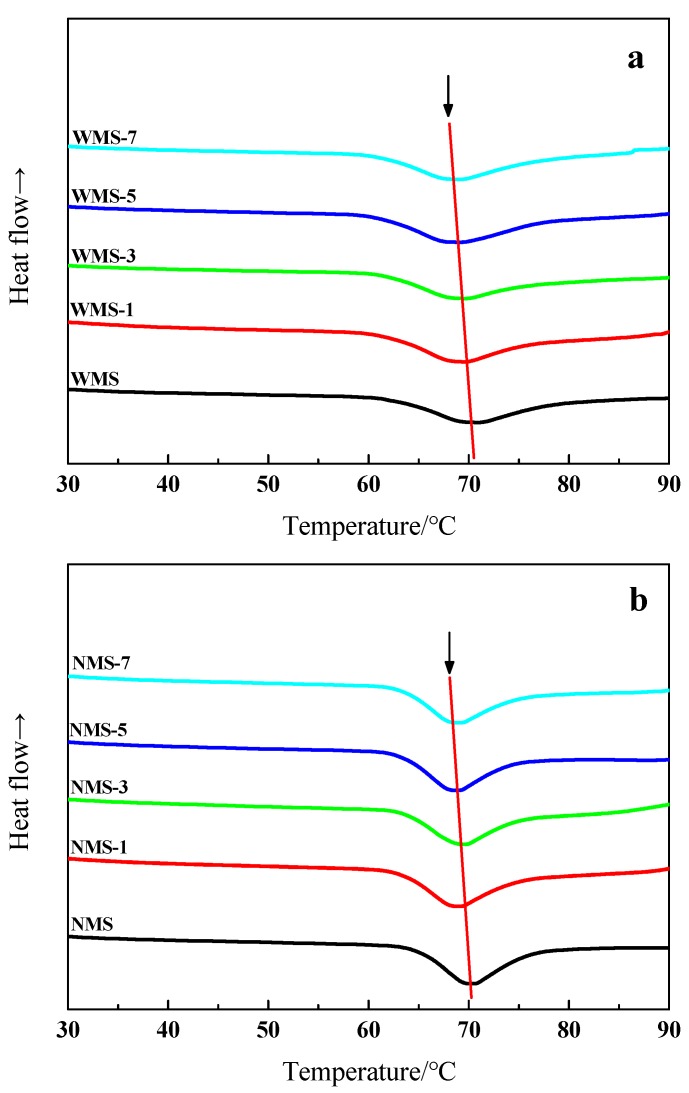
Gelatinization properties of maize starch samples treated by APPJ. (**a**) Waxy maize starch (**b**), normal maize starch.

The DSC thermograph of WMS and NMS by APPJ treatment was present in Figure 7, which the gelatinization parameters were shown in Table 2. Gelatinization parameters including onset temperature (*T*_o_), peak temperature (*T*_p_), conclusion temperature (*T*_c_), and the enthalpy change (Δ*H*). The *T*_o_ and *T*_c_ of WMS were observed to start slightly prior after APPJ treatment. Similar results were by Zhang et al. and Chen [24,35]. The changes in gelatinization temperatures might be due to the depolymerization of starch caused by the plasma species [29]. Furthermore, Δ*H* of WMS was decreased from 16.78 to 14.81 by APPJ treatment (Table 2). The Δ*H* has been reported to be influenced by the crystallinity structure of starch and granule stability [47]. The decrease in ΔH indicated that less energy was required for the gelatinization of starch [25]. Thirumdas et al. [29] and Wongsagonsup et al. [48] reported a similar decrease in the Δ*H* of rice and tapioca starches by plasma treatment, respectively. Both the depolymerization and etching caused by plasma species might result in the decrease of Δ*H* [25,29]. Meanwhile, similar results were observed in the APPJ-treated NMS. Slight decreases in *T*_o_, *T*_p_, and ΔH were found (Table 2). In summary, the gelatinization properties of both maize starches were reduced after the APPJ treatment. Notably, compared with NWS, the effect of APPJ on Δ*H* of WMS was more obvious.

## 4. Conclusions

In summary, APPJ modification as an emerging eco-friendly technology changed the structure and physicochemical properties of WMS and NMS. Plasma etching caused by plasma species resulted in slight breakage of the surface of the starch granules, which further induced changes in the functional properties, such as increases of WBC and SV, and decreases in gelatinization temperature and enthalpy. As the treatment time increased, the RC of maize starches after APPJ modification was decreased, due to the depolymerization of starch by the active species of plasma. The FTIR and Raman results indicated that the short-range molecular order was slightly reduced. This study showed that APPJ could be employed as a novel physical method for starch modification. Thus, we expect that this study will motivate further research in the field of the green production of starch products.

## Figures and Tables

**Table 1:** Structure and physicochemical properties of untreated and APPJ-treated starch samples.

Sample	pH ^†^	WBC ^†^ (%)	SV ^†^ (g/mL)	Ratio ^†^ at 1047/1022 cm^−1^	FWHH ^†^ (cm^−1^)
WMS	5.42 ± 0.06e	105.19 ± 0.20a	2.96 ± 0.04a	1.0549 ± 0.0029b	16.96 ± 0.07a
WMS−1	5.21 ± 0.02d	119.56 ± 0.27b	3.05 ± 0.03b	1.0524 ± 0.0028b	17.06 ± 0.03a
WMS−3	5.12 ± 0.03c	127.44 ± 0.43c	3.09 ± 0.04b	1.0492 ± 0.0021ab	17.41 ± 0.11b
WMS−5	5.02 ± 0.02b	127.66 ± 0.37c	3.25 ± 0.03c	1.0486 ± 0.0009ab	17.52 ± 0.08bc
WMS−7	4.97 ± 0.04a	131.27 ± 0.22d	3.33 ± 0.02d	1.0472 ± 0.0074a	17.60 ± 0.08c
NMS	5.09 ± 0.03d	83.56 ± 0.46a	2.75 ± 0.02a	1.0311 ± 0.0014b	17.44 ± 0.16a
NMS−1	5.02 ± 0.04c	86.23 ± 0.11b	2.81 ± 0.03b	1.0297 ± 0.0017ab	17.88 ± 0.03b
NMS−3	4.85 ± 0.04b	90.16 ± 0.04c	2.90 ± 0.07c	1.0265 ± 0.0078ab	17.84 ± 0.16b
NMS−5	4.78 ± 0.05a	95.82 ± 0.17d	2.93 ± 0.03c	1.0244 ± 0.0014ab	17.85 ± 0.07b
NMS−7	4.75 ± 0.02a	95.61 ± 0.27d	3.05 ± 0.01d	1.0197 ± 0.0022a	17.94 ± 0.12b

^†^ Values are means ± SD. Means with similar letters in a column do not differ significantly (*P* < 0.05).

**Table 2:** Thermal properties of untreated and APPJ-treated starches.

Sample	*T*_o_^†^ (°C)	*T*_p_^†^ (°C)	*T*_c_^†^ (°C)	Δ*H* ^†^ (J/g)
WMS	63.08 ± 0.06d	69.83 ± 0.32a	77.18 ± 0.43c	16.78 ± 0.58c
WMS−1	62.77 ± 0.46c	69.88 ± 0.06a	76.39 ± 0.30b	15.64 ± 0.37bc
WMS−3	62.37 ± 0.28b	69.68 ± 0.11a	75.38 ± 0.19a	15.52 ± 0.23ab
WMS−5	62.25 ± 0.22ab	69.70 ± 0.12a	75.40 ± 0.24a	15.23 ± 0.31ab
WMS−7	62.04 ± 0.05a	69.61 ± 0.26a	75.11 ± 0.13a	14.87 ± 0.15a
NMS	64.90 ± 0.13c	69.96 ± 0.22d	75.75 ± 0.27b	13.00 ± 0.11c
NMS−1	63.41 ± 0.14b	68.71 ± 0.25bc	74.16 ± 0.27a	12.64 ± 0.36b
NMS−3	63.37 ± 0.20ab	68.31 ± 0.09ab	74.06 ± 0.39a	12.31 ± 0.14ab
NMS−5	63.26 ± 0.06ab	68.19 ± 0.14a	73.19 ± 20a	12.26 ± 0.07a
NMS−7	63.21 ± 0.03a	68.87 ± 0.36c	73.65 ± 0.25a	12.17 ± 0.10a

^†^*T*_o_—onset temperature, *T*_p_—peak temperature, *T*_c_—conclusion temperature, Δ*H*—enthalpy.

^†^ Values are means ± SD. Means with similar letters in a column do not differ significantly (*P* < 0.05).
